# Location-aware systems or location-based services: a survey with applications to CoViD-19 contact tracking

**DOI:** 10.1007/s40860-020-00111-4

**Published:** 2020-09-24

**Authors:** H. R. Schmidtke

**Affiliations:** 19061 Schwerin, Germany

**Keywords:** Location-aware systems, Context-aware systems, Attention economy, Place, Volunteered geographic information, CoViD-19, Corona, Contact tracking

## Abstract

With the CoViD-19 pandemic, location awareness technologies have seen renewed interests due to the numerous contact tracking mobile application variants developed, deployed, and discussed. For some, location-aware applications are primarily a producer of geospatial Big Data required for vital geospatial analysis and visualization of the spread of the disease in a state of emergency. For others, comprehensive tracking of citizens constitutes a dangerous violation of fundamental rights. Commercial web-based location-aware applications both collect data and—through spatial analysis and connection to services—provide value to users. This value is what motivates users to share increasingly private and comprehensive data. The willingness of users to share data in return for services has been a key concern with web-based variants of the technology since the beginning. With a focus on two privacy preserving CoViD-19 contact tracking applications, this survey walks through the key steps of developing a privacy preserving context-aware application: from types of applications and business models, through architectures and privacy strategies, to representations.

## Introduction

When GIScience emerged as an interdisciplinary effort between computer science and geography, researchers in the area discussed the connection between technical choices, geographic perspectives, and societal consequences inherent in seemingly purely technical questions, such as the choice between raster and vector formats in GIS [35]. From the recognition that a single discipline could not solve the issues satisfyingly, the research area grew over the past four decades into a field and internationally widely established subdiscipline of both computer science and geography.

Location-aware systems today are in many ways in a similar situation as GIS technology was in the 1990s: certain systems exist and there is a rising number of societal concerns that require a deeper and more complete understanding, especially as the CoViD-19 pandemic urges for a quick response. Current applications both collect data and through spatial analysis and connection to services provide value to users. This value is what motivates users to share increasingly larger amounts of data, to “*volunteer*” geographic information (VGI) [180]. All research questions of geoinformation [70] appear again in location-aware system (LAS), when seen as a crowd-sourced spatial measurement device. For instance, questions of error-analysis, as location -aware systems are measurement-based systems [51, 66, 68, 126], or scale [69] play an important role. Consequently, the rise of citizen science gained considerably in traction when location-aware mobile applications became prevalent [174].

Many of the current technological concerns in GIScience with respect to LAS, e.g., regarding smart city technologies [108] or privacy on the Geoweb [53], are connected to the particular choice of the *location based service* (LBS) architecture currently most widely employed to realize LAS conveniently. In this conversation, the present state is one of transition in which a particular technological decision has become prevalent and is now questioned by events of societal concern regarding a too optimistic view on privacy issues inherent with the chosen architecture. Presenting prevalent and alternative solutions this article aims to broadly depict a wider angle of past and present technologies. It argues to carefully separate the two notions of LAS and LBS and proposes to replace the currently mingled usage of the terms with the following clarified definitions:

### Definition 1

A *location aware system* (LAS) is a computing system that provides services to a user-based on the user’s location.

### Definition 2

A *location based service* (LBS) is a service that answers queries that contain a user’s location information.

The main difference between the two notions is that a system being location aware is a basic question of user-centered design, a desideratum or quality of a system. In contrast, the location-based service is a specific type of computing architecture, that, *inter alia*, can be used to realize location awareness for the user. The two cannot be equated. First, a web-service that does not provide location awareness can be implemented as an LBS, e.g., to collect location data of a user. Second, there are alternative architectures for realizing LAS. That an LAS be implemented by an LBS is thus neither a sufficient nor necessary criterion, i.e., these are different notions.

The key distinction is with respect to the right to privacy [191]. The LAS criterion per se does not require users to trade in privacy. In the conventional LBS implementation in contrast, the remote web-service delivering location-dependent services requires the user to upload measured location, in order to be capable of delivering services specific to the location. This technical disadvantage additionally has an economic aspect. The web-based service architecture requires, in order to scale, a constant influx of revenue: when the web-service is down, users experience a service outage. Therefore, it has to be constantly available. For small sets of users, this is not a challenge, but for any effort that aims to operate with a large user base, Big Data, or large-scale Machine Learning (ML) technologies, considerable investments and, more importantly, constant operation costs are incurred by the centralized data center architecture required for a global web-service. The prevalent way to fund these considerable operation costs is by monetizing user data, including location data. In this business model, users exchange the ability to use the service against their location data. This trade model has been ingrained so deeply over the past decade that it has become nearly impossible to think of alternatives, and even more fundamental rights may be at stake if autonomous vehicle intelligence is funded in this manner [166].

Location awareness is the forerunner for a more general criterion of user centered response: *context awareness*, which I would like to define as follows:

### Definition 3

A *context-aware system* (CAS) is any computing system that provides services to a user based on the user’s context, where *context* can be any information about the physical environment of a user, which includes not only location and other sensory data, but also, e.g., system states or user data. Correspondingly, a web-service answering queries in return for contextual information can be called *context-based service* (CBS).

Context awareness is only touched upon in this survey with its focus on location awareness. Accordingly, a key application area of CAS, smart home healthcare is only mentioned briefly here. However, typical choices are similar in CAS, e.g.: if activity recognition is provided by a web-based service architecture for AI, where each classification is calculated by the server, users have to continuously send privacy relevant data to a server. Where, in contrast, activity recognition can be provided locally by the user’s smartphone or a wearable, this is not necessary [17].

### Use case: applications for CoViD-19

We will study a concrete use case of present concern to illustrate key aspects of developing a privacy sensitive CAS application. Going one by one through each aspect for a CAS application, we will evaluate and compare concrete suggestions for a privacy preserving solution. The most general aim is an application that allows an infected user to inform all other users they met over the past days, after he or she has tested positive for the virus. The other users, who were in proximity to the infected users, can then quickly react. Generally, it is what we would do within our everyday networks of friends: when tested positive, we would give friends we met lately a call that they may have been infected. We will use this application use case as a running example in this article. While there are many different variants and approaches, we focus on the DP3T [190] and the PEPP-PT [144], which present two of the most privacy-preserving alternatives and identify an architectural disadvantage in the use of a centralized web-server. We analyze in how far the web-server components in DP3T and PEPP-PT interfere with the primary concern of privacy protection and illustrate with the example that simple, single-purpose applications are advantageous for citizens and the environment.

From a classical GIScience point of view, LBS, rather than any other LAS architecture, resemble GIS and produce data for generating map data analyzable through GIS. Large amounts of user data when collected centrally can be transferred into GIS, analyzed with classical spatial analysis tools, and visualized with maps. User data kept by users locally are not mappable and analyzable. For CoViD-19 applications, this is a main secondary concern. Addressing both concerns at the same time is the key challenge for contact tracing applications.

### Related works

A wealth of surveys of the area exists [38, 47, 88, 111, 116, 134, 152, 153, 196]. In contrast to these, this article comes from a CAS perspective of ubiquitous computing and AI. Surprisingly, there was little interaction between the ubiquitous computing area and the LBS area after the inception of the field. Indoor location awareness and positioning approaches, for instance, were among the first applications in ubiquitous computing, and applications date back to the 1990s. LBS-focussed surveys such as [88] write in 2018: “In the early 2000s, research on LBS had been mainly focused on outdoor environments, also due to the lack of reliable indoor positioning methods, as well as the lack of indoor GIS data.” In contrast, the widely cited location system survey of Hightower and Borriello [79] from 2001, for instance, lists a range of indoor positioning technologies, such as the Active Bat with 3 cm accuracy [76] first published in 1999. This technology is also listed, e.g., in a classic textbook on location technologies in the ubiquitous computing area [119].

Huang et al. [88] list technologies mainly with a view on off-the-shelf products, e.g., depicting WiFi fingerprinting to be “the ‘state-of-the-art’ in indoor positioning,” although WiFi fingerprinting has been known to be error-prone [119]. The focus on GIS makes these technologies appear recent, as off-the-shelf products leveraging this mechanism have only recently appeared. This highlights the main issue of the mingling of the terms of LAS and LBS: as an LAS technology, these are 1990s technologies; from an LBS point of view, these technologies are 2010s technologies, as they have just recently become available as off-the-shelf products connected to a web-based server. A focus on LBS thus systematically misses technological advances that do not have the systematic privacy issues of LBS and unnecessarily makes LAS research depend on commercial viability of products under the current economical conditions. A more desirable research thrust would aim for the interdisciplinary interaction and mutual compatibility between GIScience and UbiComp groups, which may be fostered by this survey.

This survey is also what I would like to call a *historical snapshot*, an assessment of the perspective at a crucial point in time, in particular, with respect to earlier decisions that have been made. It may become relevant from a historical point of view. Three time points are particularly relevant for this survey: 1948, the year of the Universal Declaration of Human Rights [191] with its unique historical context listing privacy as a fundamental human right (article 12), 1990–2007, the time-frame within which the technologies currently commercialized were invented, and the recent past with its enthusiasm for virtualization technologies, which allow everyone access to technological advances through advertisement funding.

The development of GIS technology and a lively scientific dialogue of geographers and computer scientists show that joint approaches are possible. Accordingly, the reference selection method for this survey was chosen to capture a wide range of samples with a focus on (a) questions of privacy protection and (b) the connection to GIScience and typical GIS functionalities of analysis and visualization.

In a similar way as the computer graphics formats which implemented the first GISystems gave rise to the interdisciplinary dialogues that started GIScience and spatial information science as a subdiscipline of geography and computer science, the architectures and choices used to implement location-aware systems require a new active dialogue. Much of what, for instance, under the notion of *place-based GIS* is currently gaining traction would benefit from a local, privacy-preserving perspective of LAS as local implementations rather than global web-based services [13].

In the past decade, the market ecosystem has considerably changed as has the academic landscape with the increasing proliferation of Big Data analytics, data science, and AI algorithms [60, 78] that followed the move to an attention economy [63]. But recent events—from the Cambridge Analytica scandal^1^ to issues with LBS-based CoViD-19 tracking in the Coronaita application^2^—are putting privacy and the risks of disregard for it again at the center of discussions.

### Selection method

**Fig. 1:**
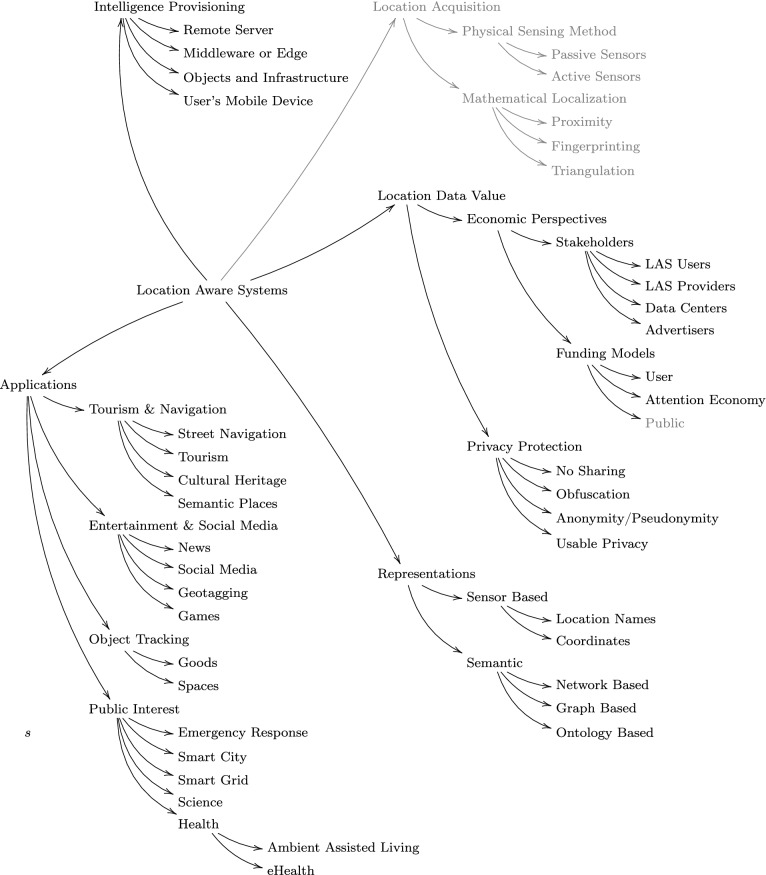
Key dimensions for classifying location aware systems

To match the above criteria and present a broad and objective perspective, especially of the fast-changing area of application research, I first performed a larger-scale Google Scholar query for the terms “location aware system” and “location based system” for the time range 2003–2018, so as to obtain a neutral sample of articles and topics (shown in Table 1). A separate sampling of articles was performed to review articles published from two journals of topical centrality for the survey, the *Journal of Location Based Services* (Table 2) and the *Journal of Reliable Intelligent Environments* (Table 3). The key dimensions used for the analysis are shown in Fig. 1.

**Table 1:** Articles retrieved from sources other than the Journal of Reliable Intelligent Environments and the Journal of Location Based Services

Ref.	Authors	Year	Citations
[2]	Abdelmoty and Alrayes	2017	7
[7]	Amin et al	2013	47
[8]	Ardissono et al	2016	15
[10]	Augusto	2007	13
[11]	Augusto et al	2013	215
[14]	Bekele et al	2018	131
[16]	Bennett et al	2008	37
[17]	Berchtold et al	2010	189
[20]	Braginsky and Estrin	2002	1961
[21]	Butz	2004	17
[23]	Catarci et al	2010	28
[25]	Chang et al	2014	324
[26]	Chen	2010	32
[28]	Chipidza and Leidner	2019	11
[29]	Choi	2007	48
[30]	Choi and Park	2015	9
[31]	Chung and Schmandt	2009	23
[34]	Coronato et al	2006	23
[33]	Coronato et al	2009	42
[38]	Dao et al	2002	138
[39]	Decker et al	2007	23
[40]	Dedecker et al	2006	194
[41]	Dhar and Varshney	2011	424
[42]	Dingus et al	2016	428
[44]	Dongqing and Xiguang	2010	5
[46]	Donohoe et al	2015	31
[47]	D’Roza and Bilchev	2003	224
[48]	Duckham	2010	36
[49]	Duckham	2012	76
[50]	Duckham and Kulik	2006	287
[51]	Duckham and McCreadie	2002	30
[52]	Egenhofer	1994	666
[53]	Elwood and Leszczynski	2011	212
[54]	Fast and Rinner	2014	53
[55]	Félix et al	2016	49
[58]	Gao and Prasad	2016	16
[59]	Gartner et al	2007	68
[63]	Goldhaber	1997	1067
[71]	Graham et al	2013	353
[72]	Gu et al	2005	1187
[73]	Haklay	2013	739
[75]	Han et al	2014	26
[76]	Harter et al	1999	2308
[74]	Hampshire et al	2016	35
[77]	Henricksen and Indulska	2006	603
[78]	Hey et al	2009	2994
[79]	Hightower and Borriello	2001	4545
[80]	Hirschfeld et al	2008	610
[82]	Hong et al	2007	33
[83]	Hornsby and Egenhofer	2000	422
[84]	Hornsby and Egenhofer	2002	341
[86]	Hu et al	2015	1101
[89]	Hupfeld and Beigl	2000	39
[90]	Hwang and Yu	2012	49
[91]	Ilarri et al	2011	34
[93]	Jacobsen	2004	23
[95]	Janelle and Goodchild	2009	42
[96]	Jang et al	2005	63
[97]	Jiang and Yao	2007	239
[98]	Jones et al	2015	34
[99]	Jordan and Klein	2020	0
[100]	Jung et al	2017	7
[104]	Kiefer et al	2012	35
[103]	Kiefer et al	2017	72
[106]	Kim et al	2009	104
[105]	Kim and Chung	2014	123
[107]	Kitchin	2014	1477
[108]	Kitchin	2014	1854
[109]	Kolomvatsos et al	2009	13
[110]	Kovacevic et al	2008	18
[111]	Krumm	2009	742
[112]	Krumm	2011	110
[113]	Kumar et al	2017	9
[116]	Kushwaha and Kushwaha	2011	184
[119]	LaMarca and de Lara	2008	91
[120]	Langheinrich	2001	1157
[121]	Langheinrich	2002	644
[123]	Leberl	2010	10
[125]	Lee et al	2017	29
[124]	Lee et al	2005	35
[126]	Leung et al	2004	124
[128]	Liu and Karimi	2006	77
[129]	Lu and Liu	2012	74
[133]	Meriste et al	2005	11
[134]	Mohapatra and Suma	2005	137
[136]	Mortenson et al	2016	21
[137]	Nadoveza and Kiritsis	2014	33
[138]	Newman et al	2017	71
[139]	Noguera et al	2012	239
[143]	Pascoe et al	1999	209
[147]	Pfoser et al	2005	41
[150]	Pradhan	2000	104
[151]	Ranganathan et al	2003	208
[154]	Rashidi and Mihailidis	2012	918
[158]	Rudin	2019	389
[159]	Salber et al	1999	1473
[162]	Schilit and Theimer	1994	2335
[161]	Schilit et al	1994	5274
[163]	Schmidt et al	1999	1581
[164]	Schmidt-Belz et al	2002	119
[165]	Schmidtke and Beigl	2011	6
[167]	Schmidtke and Woo	2009	33
[168]	Scholten et al	2009	59
[170]	Seifert et al	2007	17
[171]	Shen et al	2008	12
[172]	Shetty et al	2017	16
[173]	Shim et al	2015	50
[174]	Silvertown	2009	1837
[175]	Snavely et al	2006	3661
[177]	Steenbruggen et al	2015	179
[178]	Strang et al	2003	430
[180]	Sui et al	2012	355
[184]	Thomas	2012	103
[186]	Toch	2014	66
[187]	Toch et al	2012	216
[189]	Toyama et al	2003	468
[195]	Wan et al	2017	13
[194]	Wang et al	2015	34
[196]	Wang et al	2008	257
[193]	Wang et al	2004	1613
[198]	Wernke et al	2014	276
[199]	Wilson	2012	185
[132]	Mechael et al	2011	1609
[203]	Ye et al	2007	251
[205]	Zhu et al	2014	18
[204]	Zhu et al	2016	15
[206]	Zsila et al	2018	59
[60]	Gil de Zśńiga and Diehl	2017	39

**Table 2:** Articles from the Journal of Location Based Services

Ref.	Authors	Year	Citations
[1]	Abbas	2011	24
[3]	Aditya et al	2019	0
[4]	Alabadleh et al	2018	1
[6]	Åman et al	2015	5
[9]	Ataei et al	2018	4
[13]	Bahrehdar et al	2019	2
[18]	Bhargava et al	2015	9
[22]	Carboni et al	2015	9
[24]	Çay et al	2019	0
[27]	Chianese et al	2015	89
[32]	Coppens et al	2015	7
[43]	Dobraja and Kraak	2020	0
[62]	Gkonos et al	2017	8
[65]	Gonçalves et al	2015	9
[67]	Goodchild	2009	409
[81]	Hofer and Retscher	2017	4
[88]	Huang et al	2018	68
[92]	Jackermeier and Ludwig	2018	1
[101]	Keler and Mazimpaka	2016	23
[118]	Kveladze and Agerholm	2018	1
[127]	Li et al	2014	21
[140]	Nussbaum et al	2017	3
[145]	Perebner et al	2019	3
[152]	Raper et al	2007	151
[153]	Raper et al	2007	184
[156]	Rigby and Winter	2015	10
[179]	Stroeken et al	2015	6
[182]	Teoh et al	2019	1
[183]	Tessem et al	2015	15
[185]	Tiru et al	2010	37
[188]	Tóth	2016	19

**Table 3:** Articles from the Journal of Reliable Intelligent Environments

Ref.	Authors	Year	Citations
[36]	Crabtree et al	2018	39
[37]	Dahmen et al	2017	16
[61]	Given-Wilson et al	2018	0
[64]	Gómez-Cárdenas et al	2019	0
[87]	Hu et al	2016	29
[114]	Kumar and Mehfuz	2016	16
[115]	Kumar et al	2019	10
[122]	Le Guilly et al	2016	17
[135]	Moriyama et al	2018	1
[142]	Palade et al	2018	15
[146]	Pereira et al	2018	17
[149]	Polyzos and Fotiou	2015	22
[160]	Sasiwat et al	2020	2
[176]	Staudemeyer et al	2019	3
[202]	Yakubu et al	2019	6

A selection was made regarding specific topics. In particular, localization sensing techniques, which constitute a large portion of articles and are a necessary classification factor, were only touched upon as necessary for completeness of the remainder of the article, as sensing techniques constitute a component well-separable from the computational system. In contrast, there is not much variation on architectures and representations in newer articles. This is understandable as these topics are on the side of fundamental research requiring longer innovation cycles than applied research. Accordingly, the temporal limitation was lifted for papers from these areas.

*Caveat* The tables 1, 2, 3 list the number of citations (retrieved from Google Scholar 11–15 August, 2020), as well as author and year, giving an indication of the support enjoyed by a paper. This support is an indicator for the usefulness of the paper, e.g., to make an argument, obtain funding, etc., as the scientific community has come to critically make itself dependent on this performance indicator. However, recent scandals^3^ in science suggest caution in equating it with validity or quality. Like the number of views or likes on social media, this number will be higher: for easy-to-read papers, for older papers, for authors with higher network centrality within the social network of the paper topic, for larger social networks, for well-funded groups, for authors from social majorities, and these factors mutually reinforce each other, which can create a feedback loop effect. Whether quality generally has an influence on this metric, and whether positive or negative, would be an urgent question for future work. ICT supported science may have similar issues as are observed with social media news for society as a whole.

The structure of the article roughly follows the above analysis of dimensions. We identify four fields of research that match the focus of this snapshot survey. Research that addresses the particular value of spatial data important for business models and questions of location privacy is presented in Sect. 2. The sample of research surveyed there regards both the business models to generate revenue from spatial data and the end-user’s perception and protection of their data. In Sect. 3 we sample application research in the area and aim to outline the application research landscape within the focus. We then present main architectural choices and proposals put forth in the literature in Sect. 4 and finally aim to combine the representation choices in location-aware systems from a representation perspective in Sect. 5. The paper closes with a summary and conclusions (Sect. 6).

## The value of location data

The value of location data has been key to both users’ wish to leverage LAS and providers’ offering services in return for user location data. Two primary areas of research questions evolved from this situation:

- What is the value of location data?
  - What are the rewards users gain from sharing location?
  - What are the rewards providers gain from offering services in exchange for data?
- Concluding location data is valuable: how can users protect their data?

After a brief overview of the principle types of localization techniques in Sect. 2.1, we look at the value users and providers gain in Sect. 2.2 and at aspects of privacy protection in Sect. 2.3.

### Localization methods

A topic, which is fundamental for LAS/LBS is the question of localization technologies. While there have been recent advances, this topic is not of a primary concern given the survey’s focus. We will only briefly sketch some of the terminology and discuss research foci in so far as they overlap with the remainder of the article, i.e., as they restrict the architectural choices.

Generally, techniques can be distinguished along several systems dimensions [119], including the following: the mathematical technique, the physical foundation, the data type returned, the type of reference frame, local or global computation, accuracy and precision, scale, identification, and cost. A detailed discussion is beyond the scope of this article [cf. the textbook 158, LaMarca:2008aa]. With respect to privacy, the mathematical and physical foundations and the use of globally unique IDs for identification are most critical. Physically, sensors can be active or passive.

- Passive sensors on a user’s device receive but do not send. GPS is an example.
- Active sensors send a signal and determine location based on what they receive. Echolocation is an example.

From a privacy and location security perspective, passive sensors are advantageous.

Mathematically, there are three main techniques: proximity or coincidence, fingerprinting, and triangulation/trilateration.

- The proximity method leverages beacons, which have a known position. A receiver can then assume its own position approximately coincides with that of the beacon when it receives the beacon’s signal.
- Fingerprinting techniques are based on recognizing locations from a particular sensory fingerprint. The receiver can compare the particular fingerprint it receives with that of known locations, possibly using interpolation.
- Triangulation and trilateration leverage trigonometric laws following the idea that a triangle in the plane is completely determined if two angles and one side length is known (triangulation) or if all three side lengths are known (trilateration).

In the current literature, two main foci can be identified: first, approaches that combine different previous techniques, second, techniques for improved indoor localization with available hardware.

An example of the first category is the approach proposed by Hofer and Retscher [81]. They combine inertial sensors and compass with WiFi fingerprinting and proximity-based localization with checkpoints. On the application level, Tóth [188] collect different smartphone-based localization techniques into a single smartphone service. The crucial stage of switching between indoor and outdoor positioning techniques is the focus of two hybrid localization techniques by Teoh et al [182].

A number of research articles target indoor localization showing that it is still a focus of research with, in particular, low-cost smartphone-based approaches in focus. The error-prone Received Signal Strength (RSS) method, for instance, is targeted by Alabadleh et al. [4] with a Hidden Markov Model error correction technique. Sasiwat et al. [160] find that human movements, which will typically occur in indoor localization scenarios, results in severe fluctuations in RSS measurements and thus RSS-based localization. Moriyama et al. [135] suggest that acoustic waves may be more suitable than radio-waves. Issues in multi-story buildings are the focus of Bhargava et al. [18]. In contrast, pedestrian dead reckoning via a smartphone application is the method studied by Jackermeier and Ludwig [92]. A low-cost indoor navigation system based on maps and conventional smartphone sensors [22] promises to allow for an easy way to make public indoor spaces location aware. The approach by Carboni et al. [22] is particularly noteworthy as they make use of what is known from the location privacy perspective as a geographic attack [198]. They leverage the known geography of a location. Generally, any smartphone sensor-based low-cost method can be seen as a privacy threat highlighting that smartphones, even with location services turned off can be used for tracking by an adversary.

*Use case CoViD-19 application* In the case of the Corona applications, the critical point to track is the transmission of the disease at an earlier point in time. This can be done in a conventional temporal GIS application, if complete traces of all citizens are available. However, this method sacrifices privacy with considerable risks, as the example of the Coronaita application cited above highlighted. Analyzing the problem, we see that we do not need to track users, but only contacts between users, i.e., proximity. Consequently, both DP3T [190] and PEPP-PT [144] leverage a proximity approach. Users act as both beacons and receivers, i.e., locate their temporal snapshots of each other with respect to each other. Whenever two users come within Bluetooth Low Energy (BLE) range their devices see an ephemeral ID (EID) code the other broadcasts. Specific geolocation in the global geographic reference frame does not need to be recorded for tracing contacts.

### Opportunities from location data

The research thrust in LBS has highlighted the monetary rewards for research in LBS. A wealth of developments target designing applications through which detailed user location profiles can be obtained, analytical applications for generating user profiles for different purposes, analytical applications over large amounts of user data, and cartographic applications with which user data can be made accessible to decision makers and users. Often users have to pay a high price for sharing their information. We will only sketch some forerunners in this area and refer to surveys such as [88] dedicated to this topic for deeper enquiries regarding the financial opportunities.

As Leberl [123] notes in 2010, geodata became part of internet search as early as 2005 and location based navigation possible on every smartphone with a GPS receiver. With the proliferation of photo services, Leberl [123] envisioned a fine-grained 3D model of the world composed from smartphone photography and a field of “neo-photogrammetry” to become possible in the future. With the help of geotagged tourist photos, 3D models of landmarks had already been constructed successfully [175] in 2006.

Kim et al. [106] in 2009 discussed quality factors vital for the business value of a ubiquitous computing technology and proposed that (a) system quality measured by accessibility, stability and ease of use, (b) information quality, as measured by relevance, accuracy and timeliness of information; and (c) service qualities, measured as reliability, responsiveness, and privacy, are the major factors in the use of ubiquitous computing applications.

Jiang and Yao [97] in 2007 contrasted location aware systems and their unique properties with those of GIS. They outline as crucial research questions: positioning, location modeling, visualization and cartography, and application development.

The collection by Gartner et al. [59], forerunner of the above-mentioned research thrust in LBS, gathered results from symposia held between 2002 and 2007 on LBS and telecartography and outlines the basic characteristics of the term *location based service* (LBS) as “services, where the location of a mobile device becomes a *variable* of an information system.” The notion of LBS originated from a perspective of seeing location aware applications as an extension of applications providing maps on the internet. The mobile device from this perspective is, on the one hand, a location sensor, and on the other hand, a terminal for viewing maps provided by the server.

However, mobile devices became increasingly powerful computationally, with respect to sensors, and in their graphical capabilities. Location awareness enabled new types of business opportunities with personalized multimedia applications. Kovacevic et al. [110] showed in 2008 that geographical location awareness can be used to both increase the quality of multimedia content delivery and provide content in a more personalized way with an overlay structure for distributed multimedia communication. They highlight that this can be a means for providers to reduce costs.

While the above articles mostly study technical advantages, location information—or more precisely *proximity* information—cannot only reduce costs for providers, it also makes products more attractive and can even be considered a product by itself. A study by Jung et al. [100], for instance, found that proximity increases *social presence* for male participants in online-dating sites and resulted in increased intention to buy paid memberships. But the desire to know about the location of a potential romantic partner may be less than innocent, and women may regret that their location is monetized by the service. A study of the same year notes that privacy and security are not a priority for online dating providers [172], with major services at that time being susceptible to a simple man-in-the-middle attack.

The ability of proximity to create social presence, generally, can be exploited in a range of applications that generate profits directly through business models in the *attention economy* [63], which commodifies user attention and user data. Mobile advertisements have been proposed as important applications: Krumm [112] in 2011 hailed ubiquitous advertising as the “killer application” for ubiquitous computing. Tiru et al. [185] studied LBS in marketing and tourism management. Dhar and Varshney [41] also suggested concrete location-based advertisement applications, including promotions and coupons, presented in a given situation. They note that main advantages of the mobile devices are their small form factor and the way they accompany their user at all times, are associated with the user, personalized through user inputs, and always connected to the internet. With this amount of information, advertisements can be targeted with high precision, with advertisers willing to pay much for such fine-grained information.

The major benefit for advertisers thus is not directly in situational advertisements but rather in the collection of location data together with other personal data to create detailed profiles that can be employed in other contexts for presenting advertisements that have an increased probability to lead to action. The difference can be seen when comparing, e.g., the 2001 Google search website^4^ with a 2001 Yahoo search website^5^. While the latter search page featured numerous advertisements similar to what can be found in newspapers, each trying to attract attention, the former webpage itself shows no advertisements but lists some items as featured search results. Clicking on a link, the user provides feedback to the search engine which is collected before the user is forwarded to the respective page. The information that was collected in this way—IP of user, search terms, link clicked—is a product by itself, and the IP can be considered a virtual location.^6^

Detailed mobile location traces that allow behavioral pattern analysis became available through the mobile devices’ localization technologies and what Wilson [199] calls in 2012 users’ *conspicuous mobility*, the willingness to share behavioral location and lifestyle information with services such as Foursquare and the Twitter mobile application. In distinguishing the two types of mobile applications, we see a similar effect as with the search pages. Foursquare enrolls the user directly to produce advertisements through “checking-in” and posting the respective business to their social networks; Twitter, in contrast, appears to the users and their social networks in a way similar to a webpage where users share and discuss contents.^7^ Both types of application increase through their mobile interactivity and, by providing the opportunity for users to generate content, the chance that users find a location more memorable [199]. But even when interaction is limited, as, e.g., when reading news, users are found to prefer the mobile phone interaction [173], although one might intuitively expect a paper newspaper to offer better readability. In many cases, the actual reasons for this will be financial. Not only is the digital edition of a print medium often less expensive, there are numerous options for users to access news on the internet through paid plans or via the attention economy.

Revenue in the attention economy is in essence created by users consuming media, that is, in the search example, by the search itself as linked to information about the user. With the IP of a desktop PC, this information is limited: the PC may be shared by a family, the IP may be randomly reassigned by the provider or point to a local office of the provider. But with a smart phone the situation changes. Smart phones are not shared between users and they are equipped with a wealth of sensors. If an application is attractive to users, and users feel that it is easy and rewarding to use it to share information, the application can generate detailed profiles and thus revenue for the companies running it. This is particularly serious with social media applications. What many users and media experts do not understand is that application users are not the customers of the company developing the application, but rather that the information they provide is the product. It takes a different funding model for social media to produce social media applications that act in the interest of citizens. Only the introduction of social media applications that are not run by advertisement funding would change the situation.

The attention economy seems to generate revenue out of nothing, and it is tempting to try to somehow repair it. But it should be noted that there is a price to pay for financing applications through catching people’s attention. Not only political decision making of citizens, but also all faculties of the human mind suffer from donated attention. Attention is a highly valuable resource [157]. We may note that, e.g., after decades of falling fatalities in traffic, there was a 14% increase of deaths caused by traffic accidents within only the two years 2015, 2016^8^ with smart phone and tablet use the prevalent causes [42]. Demanding autonomous vehicles with attention economy funded intelligence should take over the streets, labeling the human mind as unreliable, is going into the wrong direction [166].

Besides the commercial interest, there is also strong scientific interest in location data traces in geography and the social sciences as Manen et al [192] note in 2009. Steenbruggen et al [177] discuss a number of spatial studies on mobile phone data demonstrating the scientific value. Public transport and associated industries benefit from location traces [195]. Such efforts, especially in urban spaces, can provide valuable insights for city management and *smart city* applications. In a more general scenario, any geographic space covered by the increasingly densely distributed mobile networks can become a *smart environment* or—when equipped with more advanced AI algorithms—an *intelligent environment* [10]: from building and campus management to globally connected applications.

*Use case CoViD-19 application* The main purpose of the applications is to enable a user who found out they are infected to notify users they were in contact with, before the disease was diagnosed. The advantage for the public is a better detection of generally or temporarily asymptomatic patients. A key factor in the spread of Corona is a patient’s infectiousness, while not yet or generally not showing symptoms. Users opt in to contribute towards fighting the disease and to benefit from faster health system response, in case they have been infected but are currently still asymptomatic. Both DP3T [190] and PEPP-PT [144] also feature a voluntary option for users to contribute data to epidemiological research.

### Location privacy

While the rising risk of public safety is a recent concern, the potential loss of privacy in CAS has been remarked early [120, 121], and alternatives are possible with dedicated ubiquitous computing technology [120]. Different architectural choices lead to different degrees of risks in privacy. A GPS signal or a stationary beacon broadcasting a geolocation does not pose any privacy risk to a mobile device receiving it. A smart phone containing map data or GIS software installed in the device or any other location-based local software does not pose any privacy risk to its user. Even running updates of maps of an area of interest, e.g., while the user is at home would, if areas are sufficiently large, does not come with any systematic risk. The location-based web-service, the transmission of software and data after submission of privacy sensitive data, is an architectural choice, and these are not the only possible choices. As we will discuss in more detail in Sect. 4, the location awareness intelligence can be installed on any of the devices involved including network components between a mobile phone and a remote service. Moreover, the choice whether to leverage a remote service or provide location awareness locally impacts not only privacy but also bandwidth.

McCrickard et al [130] note in 2009 that using dedicated technology was considered too costly for widespread use at the time. However, not only was the technology for smart network nodes or Internet of Things (IoT) devices still too costly, the web-service model offered gains instead of a cost and thus had a wider reach. Personal information is the currency in which many location based applications today are paid. Consequently, discussions on architectures for location aware systems usually center around the LBS architecture of exchange of location queries to a—centralized or, through content distribution networks, decentralized—information system. In this regard, LBS operate in a similar manner as GIS, providing access to geographical information stored in a database management system and accessed through visuospatial interfaces. The widespread equation of LAS with LBS in GIScience may be understood on this background. The main advantage of LBS, however, is not technological but financial. From a technological point of view, central collection, processing, and distribution is neither the only possible architecture nor the best. A query to a hard disk on a LAN, for instance, is answered faster and easier than an intercontinental query to a remote server.

LBS privacy research suffers from the architectural decision of making service provision dependent on the users’ submission of location information, leading to an “inevitable tradeoff” focus. Duckham [48] outlines key principles of research into LBS location privacy and highlights what makes location privacy in the model special in contrast to general information privacy, noting that, in particular, the continuity of space and the repetition of spatial patterns in time constitute specific challenges. Duckham [48] notes that location privacy proposals in the literature seldom feature a detailed attack analysis. Wernke et al. [198] realized such an analysis and classification according to attacker information. They distinguish context information, such as statistical data or geographical knowledge, temporal information, and a combination of both. A classification of the main families of approaches according to their ability to withstand the given attacks shows that considerable weaknesses exist, in particular, against an attacker able to link location data with personal data. This particular scenario is addressed with the heuristic defense technique suggested by Nussbaum et al. [140]. However, the combination of location data with personal data is a typical information collection goal for an advertisement financed service, raising further concerns regarding the LBS architecture in general. Where the eavesdropper suffers, the advertiser also does.

Duckham and Kulik [50] provide a classification of alternatives from a wider angle discussing general regulatory strategies, specific privacy policies for users, including standardization efforts, anonymity, which addresses the most dangerous type of attack identified by Wernke et al [198], and obfuscation, the intentional degradation of quality of location information. In all of these proposals, however, the architecture is an LBS architecture.

Different approaches have been suggested by LBS application developers. Coppens et al. [32] study the influence of user scripting on perceived privacy and control in location based social networks. Similarly, Ataei et al. [9] see privacy as a user-interface concern, which users can handle with more user-friendly controls. Stroeken et al. [179], in contrast, address the issue in their social network application by moving away from the conventional person -based webpages to a topic based or “place” based digital notice board, one of the most promising approaches to anonymization, and a classical LAS approach, which would not depend on a web-based service implementation.

A way to completely avoid location privacy attacks while retaining location awareness is by keeping location data and other personal data local, an approach implemented in the mobile recommender system of Han et al. [75]. This approach is increasingly attractive as smart phone processing power and network bandwidth have increased. The system runs on a mobile phone. It queries a larger number of items and then re-sorts these locally depending on detailed user information the mobile phone keeps only locally and does not transmit to the non-LBS web-service. This is a maximal obfuscation approach in the classification of Duckham and Kulik [50].

Additional concerns arise with protecting the large-scale repositories of location information that emerge with long-term trajectory collection. Lu and Liu [129] highlight that data pre-processing to protect location privacy of users is foremost an issue for the service provider side. LBS providers need to ensure meaningful services are available to customers while also making certain that the massive amounts of data so collected cannot be abused.

Given that users seem to freely share information [50], the question arises whether they are aware of the consequences. The study by Abdelmoty and Alrayes [2] indicates that the majority of users does not read privacy policies. Although awareness, especially among younger users, of typical privacy policy components is high, users are often not aware that location data, even if hidden from their social network, for instance, may nevertheless be sent to and stored at the provider if location sensing is not turned off [2]. A research area that focusses on this topic is the area of usable privacy [187]. The approach of Toch [186] uses personalization techniques to automatically generate privacy profiles from those of other users with 80% accuracy, showing that ML and Big Data mechanisms cannot only be leveraged to extract information but also to protect it.

With a wider angle on context beyond location, the ethical considerations in the development of context aware computing have been studied in the area of intelligent environments [11], with guidelines such as proposed by Jones et al. [98]. A key question is where the intelligence resides and how communication within an intelligent environment can be protected [176]. A smart home, for instance, that operates on local hardware and acts on behalf of the user rather than an external web-service, acting on behalf of advertisers, can, e.g., provide a range of AI services without requiring access to remote servers by leveraging cost-effective off-the-shelf gaming hardware [169]. Advances towards human-like AI technology may allow more efficient and more intelligent AI systems that do not require the currently massive amount of hardware required. Every human being has, even without internet access or access to a library and six to nine orders of magnitude less computational power and storage capacity, a level of intelligence that surpasses that of the currently best AI systems in versatility, flexibility, and trustworthiness. An eFriend, such as proposed by Jones et al. [98], that could answer questions and acts on behalf of its user without depending on internet access, may be a much better answer within the current AI thrust. This would be a local node architecture (Sect. 4) rather than an LBS architecture. From a GIScience point of view, cognitive GIScience at the boundary to cartography could be key for moving this technology to the GIScience domain, if new means of expression have to be identified to replace traditional map visualizations.

From a critical GIScience perspective, the main limitation in the discussion is to focus on costly large-scale cloud hosted systems considerably narrowing the choices and suggesting the “inevitable tradeoff” perspective of technology and humanity being in opposition, when in reality this is simply a question of ownership. Where the owner is the private citizen, the technology serves citizens. Where citizens do not own any devices or software, their interests are secondary to whoever owns them. Currently, the main options discussed are advertisement-funded vs. subscriber-based options [99] shifting the discussion into the virtual, suggesting cloud-hosted services to be the only option. The technical core of this is the reliance on web-services, and thus data centers, which require a constant influx of revenue to cover immense operating costs. If even more data-intensive and highly privacy-sensitive areas in the IoT, autonomous driving, and smart cities are realized as cloud services, the cost for keeping citizens secure [37, 149] and protect their privacy [36] may become prohibitive.

Generally, it is in the benefit of data center operators to seek virtualization of services to ensure upkeep and growth. But it is in the benefit of users to own the technologies they use. This may seem expensive from a naive point of view: how can a private citizen provide what Google is providing at a lower cost? However, the main costs are not incurred by the computer hardware, but by the cooling system, emergency backup power generators, security measures, etc. In the area of computing, smaller systems are easier to handle, while larger systems incur complexity overheads increasing with size [181]. A small-scale computer does not need a powerful cooling system. Also, a customer may not suffer much from not receiving social media updates during a power outage and his/her email server is probably not a target for state actors or organized crime, especially if more market diversity and increased privacy and security awareness of customers would decrease the cost-benefit rewards for criminal actors. For citizens, the security and safety measures for a private household, such as regularly running backups, could become simply a new type of regular, light household activity at the same or better security than having one’s data in the cloud.

Like people own their toasters or ovens, they could be able to own their social media provision hardware. A cloud-hosted email account costs USD 50/year, not calculating the environmental costs, whether this is paid by a subscription or advertisements. This amounts to USD 1000 over 20 years or USD 3500 for 70 years and again that much for any other cloud-hosted service. The local smart home infrastructures mentioned above, which could provide all these services in a decentralized manner, could be designed to cost USD 200–500 *once* with considerably lower environmental impact [169]. Unfortunately, we are heading into the opposite direction towards a future in which even fridges and ovens are driven by cloud-hosted services [36] with users considering an advertisement-funded vs. subscriber-based option the only viable choices. A key requirement for users perceiving alternatives will be the usability and easy controllability of personal off-the-shelf IoT packages [87, 122], a development resembling that of the 1980s thrusts towards easy-to-use personal computers with a graphical user interface.

The current security issues as well as the short life cycles of systems with minor improvements^9^ traded for increased size and lag in operation with respect to a specific device are a result of market concentration. Where a larger number of independent system producers have to agree on standards, in order for systems to communicate, while protecting their specific niches and customer base, more mature, secure, and stabile technologies result. With higher diversity moreover, targeting a specific system becomes harder and creates lower revenues for attackers. Where technology life cycles are longer, security, privacy, and social integration can be improved.

*Use case CoViD-19 applications* As discussed above, cloud hosting is a key issue for privacy. Both DP3T [190] and PEPP-PT [144] leverage a cloud-based web-service through which users communicate their infection status to contacts by uploading EIDs they received within a certain time frame prior to their CoViD-19 diagnosis. Both approaches spend considerable efforts on making these exchanges as secure and anonymous as possible. The main advantage of DP3T over PEPP-PT is that the DP3T server receives an upload of a representation of all EIDs broadcast by an infected user within the relevant time frame before the user was diagnosed. These short representations are sent to all users. Their local smart phone application then checks whether any of the time-indexed EIDs it collected matches any of the representations. As this computation is local, a connection graph of users cannot be created. The PEPP-PT architecture, in contrast, computes the infection risk of a user on the backend server. The PEPP-PT specification [144] explicitly mentions future software updates and the aim of using the data in improved epidemiological models for risk prediction as a motivation. This points at potential concerns regarding a heightened risk of *feature creep* [181] and resulting vulnerabilities in cloud-based solutions. We will come back to this when discussing architectural variants in Sect. 4.

## Types of applications

Location-aware systems have a wide range of applications. Today, many mobile applications leverage access to location information. This section gives a short sample of applications. Besides the classical application fields of navigation and tourism (Sect. 3.3), applications of public interest in governmental applications, science, and disaster management (Sect. 3.4), and entertainment and related applications (Sect. 3.2), we found that applications in object tracking (Sect. 3.1) can offer an alternative point of view on location awareness.

### Object tracking

While tracking users is a main concern with respect to privacy, tracking objects, such as business items or transportation vehicles is more neutral. While people can be tracked through their devices or other objects, a range of applications is not concerned with humans but with company assets or goods. Context awareness, e.g., has been recognized as an important factor in business process modeling (BPM) [205]. In fact, the core task of BPM could be described as a context modeling task managing the interplay between organizational and temporal context constraints in processes. The notion of context in BPM classically refers to factors such as weather, legislation, or carbon footprint, which can influence a process. However, location is classically not in the focus [205]. The study by Zhu et al. [205] motivates that location-dependency is an important factor by demonstrating its influence in previous BPM case studies. By modeling where certain steps of a process happen, constraints and potential inefficiencies in the spatial distribution of process steps can be detected, so as to be further studied in GIS [204].

When location is part of business process modeling, location tracking can be achieved in a number of ways using, e.g., smart phones or dedicated hardware for fine-grained tracking [39]. For businesses, adoption of ubiquitous computing technologies and integration of such technologies into their products or for market access depend on the overall business value that can be achieved by adopting such technologies. Kim et al. [106] show that particularly system accessibility—that is the uninterrupted availability of information—information accuracy, and timeliness, as well as quick maintenance, including service and repair after sales, are important.

A secondary area of application of location awareness regards the use of data from location-aware applications, which can be used not only for advertising purposes but also for other types of prediction applications vital for process management. Evaluation of businesses for financial prediction purposes, for instance, is particularly difficult for small businesses [194]. Wang et al. [194] showed that Foursquare check-ins allowed a significant improvement on predictive power on business failure.

The combination of classical process management and planning with location awareness is clearly a domain within the classical areas of geography and touches upon questions of logistics and operations research. Topics such as route planning or site placement optimization were classical domains for GIS. IoT supported location aware BPMs are associated with the same research questions. In fact, the integration of space and time has been a major topic of research interest for more than two decades in GIScience [16, 57, 83, 147].

With an LAS rather than LBS focus, old questions could be asked and answered differently in the variant ubiquitous computing architectures the IoT allows. Instead of harvesting data for central processing, local spatial computing applications as proposed by Duckham [49] could be leveraged. Widespread use of such IoT devices in object tracking can reduce the cost factor for ubiquitous computing applications McCrickard et al. [130] had remarked as a main obstacle. With the cost issue removed, privacy by design [120] in end-user applications could become a reality replacing the current dominance of LBS with a different LAS architecture.

BPM is an interesting LBS application, as it shows that not all LBS applications must be privacy sensitive. However, where objects are tracked through users’ mobile devices, e.g., in the ride-share industry [156], the same privacy concerns are raised.

### Entertainment and related applications

Increasingly, smart phone applications including entertainment applications are location aware. News readers today are among the most popular mobile applications [173]. An automatic news summarization that takes location into account, e.g., to be able to warn users about emergency situations nearby was proposed by Chen [26] in 2010. Location awareness has also recently been shown to increase satisfaction in online-dating sites [100], although Abbas [1] in a study in 2011 still noted that the study participants’ willingness to share location information varied depending on the amount of trust within a relationship, as would be predicted given the value of location data. Location-based music services are the focus of Åman et al [6]. Location awareness today is so widely used that many research prototypes of the past today are widely used products and successful businesses. We mention some research predecessors of today’s applications. Multimedia consumption is today an important application case [110]. Geotagging is used to index photos produced by users [189]. Users, moreover, regularly make use of local search facilities to find local businesses [29].

A major research and commercial application thrust is Augmented Reality (AR). With location awareness, large AR game worlds can be made accessible in a piecewise manner and a range of applications already exist. A place-based analysis was presented by Graham et al. [71]. Thomas [184] surveys the area from a technical perspective. A detailed psychological study of motivations to play a successful location based AR game is presented by Zsila et al [206], who argue that a loss of a sense of reality and a competitive motivation lead to problematic behavior of players. The immersiveness of AR games, their main market advantage, may lead to societal problems.

### Tourism and navigation

Navigation tools and mobile map applications are still among the most widely used applications of location- sensing technologies in mobile phones.^10^ Location awareness can support navigation tasks through the use of meaningful location models [109]. An ongoing research trend in the past decade was the addition of such semantic information, that is, the enrichment of location awareness towards context awareness. Chung and Schmandt [31] present a tool that learns the locations a user knows and presents personalized navigation instructions that require less instructions and thus reduce cognitive load for a user. Another example of an assistive location-aware navigation tool is proposed by Li et al. [127]. The system makes distant landmarks that may be occluded visible to users to support their navigation.

Semantic information from an ontology of university activities together with a 3D indoor GIS is used in the LBS proposed by Lee et al. [125]. In this application, users can ask queries rather about what they intend to do than about a specific location. The mobile GIS needs to understand the meaning of the query as a first step, before it can calculate the shortest path towards a location. Such applications for specific environments, especially where specific local web interfaces already exist, such as airport websites and services [30], constitute interesting domains for expansion of location aware technologies [106]. A hybrid tourism recommender system combining location-based collaborative filtering with knowledge-based filtering and a 3D mobile GIS for the user is presented by Noguera et al. [139]. Safety concerns of motorized tourists are the focus of [101].

A special case of navigation is given when a user’s goal is in the appreciation of sights or exhibits along the way. Museums, cultural heritage sites, and general tourism sites present this particular navigation challenge. While the problem to visit all sites can generally be considered a variant of the traveling salesman problem, many such places provide a too large number of sites to allow a visitor to visit all. Accordingly, the more specific problem is a problem of optimizing the route with respect to a number of contextual factors including factors of personal preferences or specific narratives [124] that allow a structuring of the information contents associated with each location and has been studied early as an example of an LAS application [164]. A more recent example demonstrates that not only individual navigation strategies, but also navigation of groups can be supported [61].

A range of approaches is possible in the area demonstrating application opportunities for location aware mobile applications ranging from linguistic interfaces for mobile GIS [8] to applications of eye-tracking technologies enabling extremely fine-grained attentional location awareness: *location aware mobile eye-tracking* [103, 104]. Cultural heritage exploration, however, is a domain that highlights that a web-service is not necessary from the user’s perspective. The smart environment approach using IoT devices proposed by Chianese et al [27] is a recent example. A key concern is the user-interface [62, 145]. Mobile augmented reality systems are an active field of high relevance to the area [14].

In the domain of tourism and navigation, an important focus of research from a GIScience perspective is the integration of domain ontologies into the concept of locations of relevance for an application. A museum has different types of meaningful contexts from an airport for instance, and each context is associated with a specific type of location. Whereas functional locations differ by purpose, and thus require different ontologies, other parameters of context are shared among locations, e.g., the local time of day, user data, or mobile phone system status. Generally, personalization can be improved the more a system knows about a user, the questions remain, however, (a) how much improvement a user receives with or without exchanging certain data and (b) where personalization takes place: at an instrumented exhibit that is not connected to the internet, on a user’s trusted mobile device, or on an LBS server. Applications of local use, such as guides, are an exemplary case of location aware and context aware applications that can be implemented without the user releasing location information or personal information.

### Public interests

As location awareness can help in any application that requires specific behavior at specific locations, location awareness received considerable interest from fields that operate in the public interest. One field that deserves particular attention is disaster management. We will sketch the broader landscape of past applications and then briefly discuss the special situation regarding privacy in healthcare CAS applications, including the case of CoViD-19 contact tracking applications.

The 2010 project WORKPAD [23] focussed on the collaboration between emergency-response organizations. The system combines process management and file-sharing facilities with location awareness functionality. But not only large-scale disasters can be managed better with the help of location awareness. Ubiquitous computing hardware allows the instrumentation and augmentation of everyday devices with IoT technologies so as to provide accident information. A typical project example is the car accident detection and assessment device proposed by Amin et al. [7]. The device notes the speed when the accident occurred together with the location of the vehicle. Using this information it can automatically call for help and also provide information about the potential severity of injuries of the passengers.

As a secondary application, location data collected from mobile phones have been suggested to facilitate city management towards the *smart city*. Steenbruggen et al. [177] discuss applications for mobility and transportation, for economic activities, public safety, and land-use and sustainability. Another application that deserves attention is the *smart grid*, which makes the flexible integration of distributed energy resource technologies—as occurs, for instance, in small-scale renewable energy generation through private solar panels or wind power generators—possible. Donohoe et al. [46] outline requirements and challenges for context-awareness in the area suggesting a middleware architecture.

Given how much can be inferred from user location data as discussed so far, from personal behaviors to financial risk predictions of businesses, there is a general move in the social sciences towards a location-data based approach to theory verification. The 2009 collection edited by Scholten et al. [168] discusses geospatial technologies with respect to the different sciences covering not only geography and its subdisciplines but also parts of the disciplines of archaeology, demography, economics, planning, landscape architecture, epidemiology, and criminology. While this development was only starting at that time and reluctance still existed [192], e.g., in the discipline of history, the social sciences today widely make use of Big Data approaches and tools have been streamlined to facilitate such use [25, 94, 107].

A key concern from a privacy perspective is that LBS Big Data are personal data. Where aspects of visualization are focussed [3, 24, 43, 65, 115, 118, 183], the privacy concern is generally an issue, since data need to be collected, in order to be visualized, and anonymization may not always be successful. This holds true whether the data are collected by businesses, governments, or grassroots initiatives, such as in citizen science and NeoGeography [67, 73]. LBS are attractive in so far as they are easy to implement as global web-services with GPS-enabled mobile phone applications. The study by Newman et al. [138] explains citizen science as “place-based” and “built on in-person participation.” IoT devices operating locally can realize this ideal in a privacy preserving manner without global web-services.

A wealth of applications exists in the healthcare area, so many, in fact, that there are several surveys dedicated to subareas. Particularly prevalent areas are mobile health (mHealth) scenarios leveraging LBS [132] and ambient assisted living (AAL) scenarios with IoT technologies [154]. It should be emphasized that, in terms of privacy concerns, the health area is a particularly sensitive area. On the one hand, privacy of health data underlies the strictest constraints, as abuse has the most severe consequences [120]; on the other hand, people suffering from a disease are often ready to release their data out of desperation. AAL scenarios often involve a dystopian level of surveillance [136], and elderly people, afraid of a fatal situation or a relocation into a nursing home, will agree to almost any sensor installation. The situation is similar for mHealth. The cost for mHealth in ICT for development (ICT4D) scenarios, for instance, is often borne by community health workers (CHW) [74], volunteers serving poor local communities without compensation.

*Use case CoViD-19 applications* The desperation caused by the fear of death is also among the greatest concerns regarding CoViD applications. For the Corona applications, two primary concerns collide: the desire to, on the one hand, deliver a privacy preserving contact tracking and risk alert functionality to citizens, and, on the other hand, to provide decision-makers in the health sector and researchers in epidemiology with insights into the spread and distribution of the disease. It generally holds that where data is collected, it can be stolen or abused. DP3T is stronger on the side of citizens, as its privacy mechanism is stronger. The PEPP-PT web-service, in contrast, is designed so as to allow more flexible experimentation with different models. Both platforms make it voluntary for users to donate additional data to help with epidemiological research.

## Architectures and implementation variants

Location aware systems, such as the location aware news summarization tool by Chen [26] or the disaster response coordination tool by Catarci et al. [23], which are written with a conceptualization of location-aware systems as extending web-services, are developed using an architecture resembling that of web-services. As we argued, the notion *location based service* (LBS) is usually understood to have this meaning. Location aware systems (LAS) comprise of a wider range of architectural choices, with the web-service architecture being only one of several, and in a strict sense refers only to the system’s ability to react in a relevant way differently in different locations, a behavior which can be implemented in various ways. Butz [21] frames the main question: “Between location awareness and aware locations: where to put the intelligence”? Computational resources in location aware systems are contributed by three types of devices [21]: (a) the user’s mobile device, (b) devices installed in the environment—including ubiquitous computing and IoT devices, but also mobile operators’ infrastructure devices—, and (c) remote servers. The perspective of LBS as web-services puts most of the load on the remote server (c), which was understandable given the computational resource limitations of earlier cell phones and IoT hardware.

A range of proposals suggest to deploy local middleware platforms [142, 146] operating within a certain area and relieving burdens in IoT deployment, control, and usability. In a similar vein, current developments in 5G infrastructure push computational resources closer to the user to free network bandwidth and reduce round trip time, a strategy called *edge computing* [86] or *fog-computing* [64, 202] which could also be used to bring LAS as distributed services closer to the user. The early CRUMPET framework proposed by Schmidt-Belz et al. [164] integrated interfaces for service provision, e.g., web-based search for restaurants or local events, with interfaces for local GIS servers. Further components were environmental modeling, user modeling, and a mediator module relating between the modeling components and the client agent component running on the user’s mobile device. All the basic components of LBS, if not at the scale of today, were already present in this framework together with an intermediate agent software representing the user’s interest.

Special application scenarios, in particular disaster management, suggest more flexible or complex architectures for establishing location involving heterogeneous and ad-hoc network solutions including, e.g., technologies such as MANETs (mobile ad-hoc networks) [113, 114] or sensor integration [90]. A localization method based on spatial analysis with GIS was proposed by Gao and Prasad [58].

Not only the provision of the services but also the location sensing is contributed from a variety of sources [41]: satellite-based location tracking, sensors and RFID in the environment, location management on the side of cellular service providers, and identification from localization of WiFi Access Points.

Location awareness, like context awareness in general, can be realized in different ways. From a program execution perspective, context aware control or triggering of functionality means introducing a branch into code execution. Code branching in multiple locations is, due to its many execution alternatives, harder to read, debug, and verify than code that has a smaller branching factor. One way to handle this particularity is the use of a context model, which we discuss below (Sect. 5). When context aware systems are viewed from a software engineering perspective as stand-alone systems, alternative behaviors can be implemented using patterns and facilities provided by the programming paradigm.

An object-oriented approach was proposed by Pascoe et al. [143]. Their Stick-e Note message board features an environment module for maintaining contextual information, a triggering engine that repeatedly performs queries over the current context and activates responses when a query is fulfilled, and a manager that connects client applications to the framework.

The solution proposed by Oliveira et al. [141] suggests the use of Aspect-Oriented Programming (AOP, Kiczales et al. [102]) to make Web-GIS development more flexible. In AOP, *cross-cutting concerns*, such as access control or logging, that are required to be performed within functions but that do not have a relationship to the function’s actual purpose, e.g., modifying information in a database, can be *weaved into* the code to improve clarity and separation of concerns. As context information modifies code in various manners the approach proposed by Hirschfeld et al. [80] extended the aspect-oriented paradigm into a context-oriented programming paradigm. Another dedicated approach extending the object-oriented paradigm was proposed by Dedecker et al. [40].

An agent-oriented approach [201] is used by [133, 164]. Meriste et al. [133] suggest an agent architecture for location aware and time-aware participatory GIS featuring agent components for information management, map agents for preprocessing map fragments, spatial agents which are activated on the map view, agents for handling thematic information, and location agents that provide location-tracking.

The agent-oriented approach belongs to the larger category of AI approaches to circumvent the difficulties of handling code with a large number of branches. Machine learning (ML) is another approach within AI that can be used to overcome this challenge. An ML-based LAS has a simple program structure with most or all of the decision making code being automatically generated by a process called *training*. While an introduction into ML is beyond the scope of this article [cf., e.g., 158, WittFran.2000], the simple example of a particularly simple mechanism may serve to illustrate the method. *Decision Tree* learners, such as C4.5, generate decision trees based on statistical properties of *annotated training data*, data that have the same format as the later input data of the system, and for which the desired output is known. The decision tree consists of a series of nested if-then conditions, i.e., provides a mechanism not only to autogenerate branching code but also to encapsulate it. The autogenerated code is called a *classifier*. It can be embedded in a location aware application as a *black box*, into which the application programmer does not have to look, similar to program parts imported from external modules. Confidence in the classifier comes through testing it with *test data* and checking statistical measures of confidence, such as, e.g., the rate of true-positives. The information hiding and convenience the black box provides, however, is increasingly being criticized [e.g. 158] as societal damages have begun to surface. Where, for instance, training data are racially biased, a supposedly objective algorithm perpetuates or even solidifies racial discrimination. As training data and test data are necessarily data from the past, one must be prepared to see decision making that repeats decision making following overcome ideas.

Another important part of location aware systems engineering not mentioned above is the user interface. To give just one example: McCrickard et al [130] propose an approach combining agile software development and usability engineering, the agile usability approach.

**Fig. 2:**
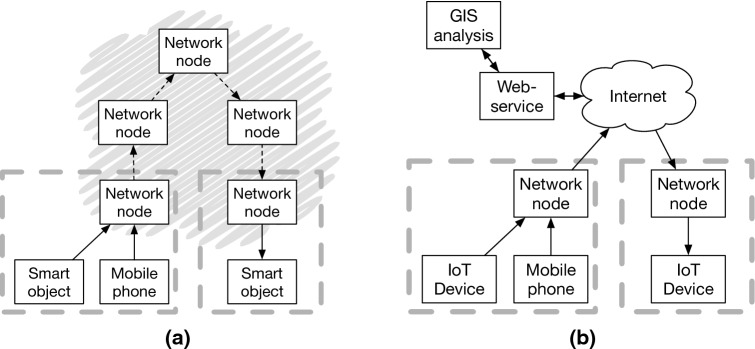
Architectural variants of location-aware systems: **a** classical network-based LAS with distributed spatial intelligence, **b** LBS as LAS implemented as a web-service

Architectural decisions as to the way location-aware systems can be realized are currently mostly made under economic perspectives with an eye on maximal data collection needed to create the continuous revenue stream required to sustain data center operation costs within the attention economy paradigm. Security, safety, and privacy are often only secondary concerns. Given the wide variety of potential alternative location aware system architectures, it seems likely that a wider diversity of approaches could be developed. Figure 2 contrasts the two extreme cases in networked LAS: location-aware provision of services as well as analysis can (a) be provided by the network and distributed AI approaches, or (b) be centralized on the side of the web-service, the currently prevalent LBS model. Earlier LAS approaches and the latest edge computing are closer to the model in (a). Not shown are approaches that run without network access to establish location awareness, e.g., leveraging GPS only and running on a user’s personal device, such as a smart phone [e.g., 75]. Agent-based approaches provide fine-grained control to programmers over where data are processed. Smart home agents, for instance, could be implemented in such a way as to be disconnected from the internet. With current AI technology, however, relying on increasingly larger data repositories, user agents today require internet access for intelligence. But this is, again, not a principle necessity but a choice suggested by the paradigm: a human being has better intelligence with lower hardware and storage requirements, and conventional low-cost entertainment systems contain the necessary hardware to run ML training [169], which personalized to a specific set of users [17] could even offer better recognition rates.

*Use case CoViD-19 applications* Coming back to the initial motivation to allow a user diagnosed as Corona positive to notify users he may have infected while being asymptomatic, we can see that both DP3T [190] and the PEPP-PT [144] leverage a web-service approach for delivering notifications. But while PEPP-PT additionally performs the risk assessment on the web-service, DP3T computes it on the user’s trusted device. In order to find even better alternatives, decentralized notification architectures could be leveraged. While a pure peer-to-peer approach, such as rumor routing [20], suffers from large overheads, intermediate approaches, such as edge or fog computing [64, 86, 202] or personal smart home infrastructure, could be developed to offer distinctive advantages in terms of privacy, network and server load, and environmental sustainability.

## Modeling and representation

A fundamental question for location aware systems and, more generally, context aware systems is how to represent location or context, generally. When the notion of *context awareness* was coined [161, 162] as part of the idea of *ubiquitous computing* [197], the notion of *context* employed was foremost one of sensor values, including location, light intensity, devices nearby, or applications running, that could be used to trigger behaviors, both from a device and devices around it. Networks in these early systems played an important role [89] and with simple comparison operations [159] the network hierarchy allowed an efficient representation of hierarchical reasoning about location and sensor information. Early, the need for adding semantics to the IDs, called *symbolic location*, or coordinates, called *physical location*, obtained from sensors was realized. If the unique IDs of URLs are used as IDs, arbitrarily complex semantical information can be linked to locations [91, 150]. Increased application flexibility could thus be achieved by viewing information on the background of a description of the world around an application. These representations of the world around a device are called *context models*—or, where the focus is on location only, *location models*—and efficiency of the overall system is a main criterion as application triggering and behavior adaptation have to be activated in real-time or near-real-time to guarantee timely reaction.

These early light-weight context aware systems already had to manage sensory uncertainty, ad hoc network aspects, software engineering aspects, inference and reasoning, human-computer interaction, and privacy handling. Context modeling approaches differed as to the question on which aspect they focus, e.g.: networks [161], sensor-fusion [163], or context aware application software engineering [77]. Ensuring interoperability between heterogeneous localization techniques and context models integrated in different environments and the switching between environments moved into focus. What is desirable behavior for a mobile phone in a classroom or supermarket differs considerably [44], and a smart office [34] has different information provisioning requirements from a smart hospital [33]. The de-facto standard that emerged were ontology-based approaches [72, 151, 178] connecting the previous more network-centered notion of context awareness to the established formalisms of the knowledge representation area, in particular, to the description language formalisms [12] in the KL-ONE tradition [19] that constitute the de-facto standard in web ontologies through the web ontology language OWL [85, 131]. The advantages of this standardization process are particularly in interoperability: ontologies about a certain domain can be used in any application in the domain [105, 137].

However, representing and reasoning about knowledge within the main areas of context, in particular time and space, is not a focus of standard OWL [203]. Consequently, ontology-based context modeling approaches often additionally leverage logical languages more expressive than OWL [72, 151, 178], e.g., for representing rules [34] or space and time [151]. Research in the qualitative reasoning area, however, demonstrated that temporal and spatial knowledge can be represented and reasoned about in a tractable manner [155]. Moreover, Egenhofer [52] notes that usability can be higher with qualitative than with quantitative interfaces to spatial knowledge. A particularly light-weight qualitative spatial reasoning (QSR) mechanism for distributed spatial reasoning on MANETs was proposed by Schmidtke and Beigl [165]. The example application scenario shows how a knowledge-based traffic information system among cars moving along a highway can be established without a web-service or centralized data collection and a minimal hardware configuration.

A particularly active field within the knowledge representation area is focussing on developing specialized logical languages for specific domains that can provide tractable reasoning. Description logics, the formalism underlying OWL, for instance, were developed with a focus on representing and reasoning about taxonomic concepts and relations [45]; spatial reasoning was the focus of the logics studied by Bennett [15]. When specialized tractable logics are combined, the resulting logic has to be studied in detail and may have the same [117] or higher complexity [56]. A specialized logic called *Context Logic* originated from discussions [82] regarding how to imbue the sensor based 5W1H context model of Jang et al. [96] with tractable reasoning from QSR research. It underlies the distributed QSR mechanism of Schmidtke and Beigl [165]. An ontology-based verification method for context-aware systems based on this logic was proposed in [167].

Especially with respect to rich domain ontologies, such as the campus activities of Lee et al. [125], an ontology-based approach is advantageous. The ontology of Lee et al. [125] is based on the ontology proposed by Wang et al. [193]. A GIS-oriented ontology for LBS is proposed by Shen et al. [171] and combines OWL with the concepts employed in the geography markup language (GML^11^) developed by the Open Geospatial Consortium (OGC).

In contrast, the publish-subscribe middleware by Jacobsen [93] employs a model of subspaces similar to that employed in [167].

When viewed collectively from a GIScience perspective leveraging the classification of Janelle and Goodchild [95], one may note that spatial representations in the above classical LAS encompass location, neighborhood and region, and partially scale, but leave out aspects of geographic networks, spatial heterogeneity, spatial dependency, and objects and fields. By modeling moving objects depending on their speed as spatio-temporal cones the potential influence relations between objects can be inferred, an approach introduced into GIScience by Hornsby and Egenhofer [84]. Statistical and ML approaches are used in Liu and Karimi [128] to establish location awareness using a network model to predict trajectories. Since then, a number of ML approaches have been proposed that would deserve their own survey, such as for using deep learning for extracting critical locations in WiFi Fingerprinting [55].

Seifert et al. [170] study spatial representations for an interactive spatio-temporal problem solving assistance system from a cognitive and AI perspective. They note that the computational problem if attempted without pre-structuring would be too difficult and propose an approach to leverage granularity hierarchies.

As a domain of central concern for GIScience, the representation of location in its different appearances within location aware systems, e.g., personal locations, traces, relative and absolute locations, location hierarchies, quantitative and qualitative, is a core concern of GIScience. Ubiquitous computing research rediscovered many of these aspects, GIScientists have an important role here to contribute their expertise to this discussion, so as to find optimal representation mechanisms.

*Use case CoViD-19 applications* In the case of the CoViD-19 application scenario, the main information for tracking is the list of EIDs identifying contact events. Both DP3T and PEPP-PT are strong with respect to privacy, as they track contact events, not user IDs, as a conventional LBS would, together with coarse time of day information. The representation of space for contact tracking is thus local to the moving reference frame of the user.

## Conclusions

This survey discussed location aware systems 72 years after the Declaration of Human Rights, which on the background of the previous abuse of, inter alia, location data declared privacy a human right in 1948. Especially, the mobile area has changed considerably over the past decade with perspectives changed by LBS and social networks. For many, the Cambridge Analytica case^12^ became a warning sign that we have become too careless about trading our privacy for enjoying services, questioning the sustainability of the entire advertisement-based financing model and thus the long-term sustainability of the web-services architecture. A more *humane* technology is sought even by designers and application developers from within the attention economy.^13^ The current CoViD-19 crisis, moreover, rekindled discussions regarding the LBS model and highlighted the political dimension with some nations leveraging citizen tracking applications while others are opting for citizens having maximal privacy and control over data with more secure contact tracing applications. As a running example, we discussed the DP3T and PEPP-PT models, which were designed with optimal privacy protection as a core value and, although leveraging a central server for broadcasting messages, apply a simple proximity model of location that aims to minimize use of the web-service to ensure privacy protection.

However, already a counter movement is underway, with Piller [148] arguing prominently in the journal *Science* that privacy, which he renames “data secrecy,” costs lives and that relaxed privacy legislation in the face of human suffering should be leveraged to push for a quick cure, an argument familiar from discussions on autonomous vehicles in the context of traffic fatalities [166]. The proof that this is not the case is given by the two privacy-preserving contact tracing applications discussed in this paper and the fact that other countries achieved to bring the pandemic under control without delimiting citizens’ rights. The inability of the US to tackle the pandemic has its roots not in technical impossibility or the need to get access to citizen health data but in cultural historically rooted specificities and a preference of personal pleasure and convenience over public interest.

Where Germans’ initial reaction was to buy face-masks worried to protect the elderly and people with preconditions, a practice that was already widely in use throughout East Asia long before the pandemic, a first reaction in the US was to see the mask as a symbol of oppression, resisting the mask for continuing a pleasure-oriented lifestyle, as resisting oppression. A cooperative, serious perspective out of compassion worried to harm others, especially the elderly, is unthinkable for some.^14^ This trend then spread to other countries, where similar movements have now started to threaten the early successes. As CoViD-19 is predicted to not have been the last pandemic under climate change [5], decisions made now may have a far reach. For the health of the population, democracies, and human rights, it is to be hoped that the international community follows a rational, situation-adapted model.

Mechanisms for contact tracing like DP3T and PEPP-PT constitute a technological population immunization mechanism. The relative localization mechanism already works without interaction with a server. Replacing the central server with a partially decentralized architecture and a trusted, smaller-scale operator, such as a local government agency, would be a step to further increase usefulness of the systems and even produce mappable data for medical purposes without sacrificing trustworthiness, and at a much lower carbon footprint. If cities offer public terminals that can be used to send and receive messages to pseudonym IDs, the communication can be completely anonymized while still offering location data at the level of granularity of the terminals. This would be a way to generally provide anonymous, trustworthy, and easy to use location-aware systems in a post-attention economy that is more worried about protecting the planet and humanity than about continuing a pleasure-oriented lifestyle.

## Footnotes

1 www.theguardian.com/news/2018/mar/17/cambridge-analytica-facebook-influence-us-election

2 http://www.nytimes.com/reuters/2020/05/14/world/asia/14reuters-health-coronavirus-southkorea.html

3 https://www.theguardian.com/world/2020/jun/03/covid-19-surgisphere-who-world-health-organization-hydroxychloroquine

4 https://web.archive.org/web/20010504033755/http://www.google.com:80/

5 https://web.archive.org/web/20010503173725/http://www.yahoo.com:80/

6 With limitations in accuracy, IPs can inform about physical location as well. Providers register IPs with a location that can be inspected using services such as https://www.iplocation.net.

7 The distinction between active and passive VGI as discussed by Fast and Rinner [54] comes to mind in this context. However, Foursquare could be designed as an LAS with a privacy preserving non-LBS architecture (see Sect. 4).

8 https://www.nsc.org/NewsDocuments/2017/12-month-estimates.pdf

9 Apple suggested when introducing the then new operating system Mojave in 2018, that the new “Dark Mode,” a minor, optional, visual variation of the interface for working at night, was a main improvement. With another UI mode to support, software development costs are increased. With a smaller number of developers, such changes can impact smaller companies’ competitiveness.

10 US Android market reach 96% according to https://www.statista.com/statistics/243763/us-market-reach-of-the-most-popular-android-smartphone-apps/

11 https://www.opengeospatial.org/standards/gml

12 https://www.theguardian.com/news/2018/mar/17/cambridge-analytica-facebook-influence-us-election

13 https://www.humanetech.com

14 https://www.theguardian.com/world/2020/mar/24/older-people-would-rather-die-than-let-covid-19-lockdown-harm-us-economy-texas-official-dan-patrick
